# Clinical Differences Among Histological Categories of Sarcoma: Insights from 97,062 Patients

**DOI:** 10.3390/cancers17101706

**Published:** 2025-05-20

**Authors:** Yiqun Han, Ahmed Shah, Yuan Yao, Robert W. Mutter, Meng Xu-Welliver

**Affiliations:** 1 Department of Radiation Oncology, Mayo Clinic, Rochester, MN 55905, USA; han.yiqun@mayo.edu (Y.H.); mutter.robert@mayo.edu (R.W.M.); 2 Department of Pathology, Mayo Clinic, Rochester, MN 55905, USA; shah.ahmed@mayo.edu; 3 Department of Oncology, Mayo Clinic, Rochester, MN 55905, USA; yao.yuan@mayo.edu

**Keywords:** sarcoma, histology, heterogeneity, overall survival

## Abstract

**Objectives:** To evaluate the clinical heterogeneity of sarcomas by examining associations between histological subtypes, metastatic patterns, treatment modalities, and survival outcomes. **Methods:** We analyzed data from 97,062 adult patients diagnosed with sarcoma between 2000 and 2020, using the Surveillance, Epidemiology, and End Results (SEER) database. Fourteen histological subtypes were included. Propensity score matching (PSM) was employed to adjust for baseline differences, and Cox proportional hazards models were used to identify prognostic variables. **Results:** The most prevalent subtypes were sarcoma not otherwise specified (31.9%), leiomyosarcoma (17.1%), and liposarcoma (13.9%). Metastatic patterns differed significantly by subtype; liver metastases were most common in sarcomas with small blue round cell (SBRC) features (8.9%) and stromal sarcoma (6.1%), while lung metastases were frequently observed in Ewing sarcoma (10.0%) and rhabdomyosarcoma (9.7%). Median overall survival (mOS) varied widely, ranging from 234 months in chondrosarcoma to 16–20 months in rhabdomyosarcoma and SBRC sarcoma. Overall, patients with primary sarcoma had significantly better survival than those with treatment-related disease (119.0 vs. 45.0 months, *p* < 0.0001), with this trend consistent across most subtypes. Treatment responses were subtype- and size-dependent. For instance, surgery plus radiotherapy improved outcomes in giant cell sarcoma regardless of tumor size, whereas chemotherapy provided benefit only in tumors larger than 5 cm. Combined surgery and radiotherapy offered additional survival benefit in select subtypes, including chordoma, leiomyosarcoma (>5 cm), and synovial sarcoma (<5 cm). **Conclusions:** Sarcomas exhibit substantial clinical and prognostic heterogeneity across histological subtypes. These findings underscore the importance of subtype-specific, individualized treatment strategies in optimizing patient outcomes.

## Simple Summary

Sarcomas are a rare and diverse group of cancers with considerable variation in clinical behavior depending on their histological subtype. Despite this heterogeneity, treatment approaches often remain generalized. In this study, we analyzed data from over 97,000 patients to identify key differences among 14 sarcoma categories, focusing on patterns of disease metastasis, overall survival, and treatment outcomes. Our findings revealed substantial variation in metastatic behavior and prognosis across subtypes, as well as differential responses to surgery, radiation therapy, and chemotherapy. These insights highlight the need for more individualized treatment strategies and may inform clinical decision making and future research efforts aimed at improving outcomes for patients with sarcoma.

## 1. Introduction

Sarcomas represent a rare and biologically diverse group of malignancies originating from mesenchymal tissues, accounting for approximately 1% of all adult cancers and up to 15% of pediatric solid tumors. They encompass both soft tissue and bone sarcomas and are characterized by substantial heterogeneity in terms of histological features, anatomical origin, molecular alterations, and clinical behavior. This diversity often leads to variable disease progression, treatment response, and survival outcomes among affected individuals [1]. About 13,590 new cases of soft tissue sarcoma and 3970 new cases of bone sarcoma will be diagnosed in 2025, with 7250 estimated deaths [2]. Despite advances in surgical techniques, radiation therapy, and systemic treatment, the overall prognosis for sarcoma patients remains unsatisfactory, particularly in cases of metastatic or recurrent disease. One of the key barriers to improving outcomes lies in the complex and multifaceted nature of the disease itself.

Currently, more than 70 distinct histological subtypes of soft tissue sarcoma have been recognized by the World Health Organization, in addition to numerous bone sarcoma variants and sarcomas arising from specific organs [3,4]. Each subtype exhibits unique biological behavior, which influences clinical decision making and therapeutic response. For instance, while some subtypes such as gastrointestinal stromal tumors (GISTs) have well-established molecular targets and treatment algorithms, many others—particularly rare or poorly differentiated subtypes—lack specific therapeutic options and remain understudied. Moreover, due to the rarity of individual subtypes, clinical trials and retrospective studies often focus on a limited number of histology samples or are restricted to single-institution experiences [5,6,7]. These constraints hinder our ability to form a comprehensive understanding of sarcoma as an entity. Although histology-driven approaches are increasingly being adopted to advance precision oncology, a major challenge persists in stratifying patients accurately due to overlapping features and inconsistent classification standards. Furthermore, the clinicopathological correlates and prognostic implications of different histological subtypes have not been systematically evaluated on a population level. Without large-scale, data-driven analyses, clinicians are often left with limited evidence to guide subtype-specific treatment planning and risk assessment.

In light of these challenges, we conducted a large-scale, population-based study to evaluate the clinicopathological characteristics and survival outcomes associated with various histological subtypes of sarcoma. By analyzing data from a nationally representative cohort of 97,062 sarcoma cases, our objective was to identify patterns of heterogeneity and uncover clinically relevant insights that could inform histology-specific management strategies. Through this work, we aim to contribute to a deeper understanding of sarcoma biology and support the development of individualized therapeutic approaches in real-world clinical practice.

## 2. Materials and Methods

### 2.1. Population

The sarcoma dataset was retrieved from the Surveillance, Epidemiology, and End Results (SEER) database (2000–2020). Individual datapoints were collected and underwent selection and eligibility evaluation. The inclusion criteria were as follows: (1) ≥18 years of age; (2) definite histological subtypes; (3) histological subtypes confirmed by positive histology, excluding diagnoses based solely on imaging, cytology, or clinical suspicion. Patients were excluded if population demographics and clinicopathological features were incomplete. Clinical variables in this study consisted of age at diagnosis, sex, race, first malignant primary indicator, laterality, histological grade, tumor size, localized lymph node metastasis, metastases (bone, brain, live, lung), surgery, radiation therapy, and chemotherapy. The American Joint Committee on Cancer (AJCC) 6th guideline was utilized for TNM staging of sarcoma [8].

### 2.2. Outcomes

**Figure 1:**
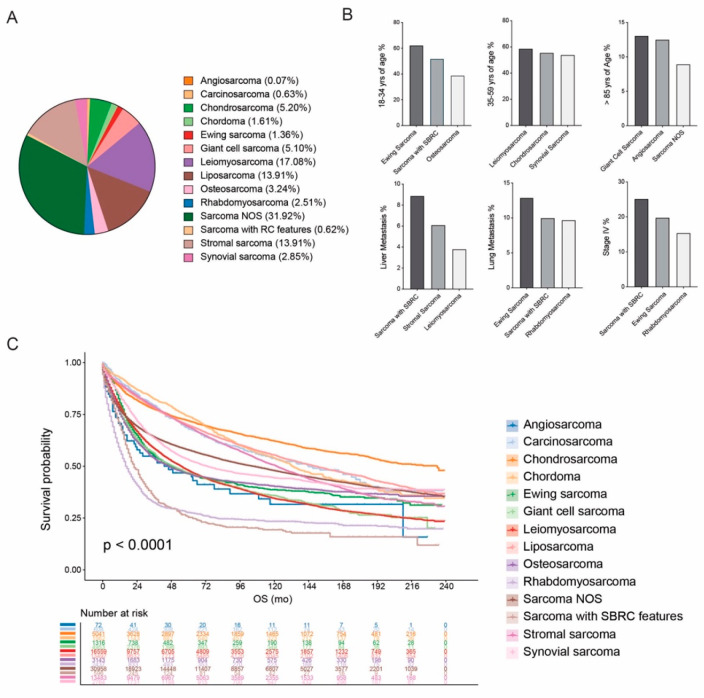
Clinical features and survival outcomes of sarcoma with different histological subtypes. (**A**) The proportions of 14 histological subtypes of sarcoma in this study. (**B**) Featured characteristics at baseline of histology-based sarcoma. (**C**) Survival outcomes of sarcoma with different histological categories.

In this study, the histological subtypes based on the ICD-O-3 codes of each patient were extracted. We summarized and classified the categories recorded in the SEER database into 14 histological subtypes according to the clinical behaviors and features, including angiosarcoma, carcinosarcoma, chondrosarcoma, chordoma, Ewing sarcoma, giant cell sarcoma/undifferentiated pleomorphic sarcoma (UPS), leiomyosarcoma, liposarcoma, osteosarcoma, rhabdomyosarcoma, sarcoma not otherwise specified (NOS), sarcoma with small blue round cell (SBRC) features, stromal sarcoma, and synovial sarcoma (Figure 1). Round cell liposarcoma (previously considered a distinct subtype) was grouped under the liposarcoma category, reflecting its classification as a high-grade variant of myxoid liposarcoma. Liposarcoma, NOS cases were also included in this group. Sarcoma not otherwise specified (NOS) refers to tumors coded using ICD-O-3 histology terms that lack defining features to be assigned a more specific subtype. In our study, this category included spindle cell sarcoma (8801/3), epithelioid sarcoma (8804/3), undifferentiated sarcoma (8805/3), fibromatous sarcoma (8810/3–8815/3), myofibroblastic sarcoma (8825/3), malignant fibrous histiocytoma (8830/3), and myxosarcoma (8840/3). These codes represent tumors with ambiguous or overlapping morphologies often diagnosed in the absence of molecular or immunohistochemical confirmation. Sarcomas were categorized as “primary” or “secondary” based on the SEER variable “first malignant primary indicator”. Cases marked “no” were considered secondary sarcomas, indicating that the sarcoma was not the patient’s first primary malignancy. Overall survival (OS) was defined as the duration from diagnosis of sarcoma to the death caused by any reasons or the last follow-up.

### 2.3. Statistical Analysis

Comparative analyses of clinicopathological features of sarcoma with different histological subtypes were conducted using Pearson’s chi-square and Fisher’s exact probability tests for qualitative data and *t*-tests or Wilcoxon rank tests for quantitative data on normal and abnormal distributions, respectively. Propensity score matching (PSM) analysis was performed to calibrate the objective differences in variables at baseline of sarcoma subgroups. Survival outcomes were compared using the Kaplan–Meier method with log-rank tests. Prognostic factors of sarcoma with different histological categories were identified with the univariate Cox proportional models first, of which the significant variables were added to the multivariate Cox regression analysis to be assessed for independent prognostic factors of each histological subtype. All statistical analyses were two-sided and *p* < 0.05 was considered statistically significant, and analyses were performed by R software (3.6.3).

## 3. Results

### 3.1. Patient Cohort and Histological Categories

A total of 97,062 patients diagnosed with sarcoma were identified from the SEER database (Figure S1 and Figure 1A). Based on clinical characteristics and histological behavior, sarcomas were classified into 14 distinct categories, including: angiosarcoma (72, 0.07%), carcinosarcoma (608, 0.63%), chondrosarcoma (5043, 5.20%), chordoma (1562, 1.61%), Ewing sarcoma (1316, 1.36%), giant cell sarcoma/UPS (4953, 5.10%), leiomyosarcoma (16,574, 17.08%), liposarcoma (13,505, 13.91%), osteosarcoma (3144, 3.24%), rhabdomyosarcoma (2439, 2.51%), sarcoma NOS (30,980, 31.92%), sarcoma with SBRC features (596, 0.62%), stromal sarcoma (1305, 13.91%), and synovial sarcoma (2765, 2.85%). Detailed clinicopathological characteristics are summarized in Table S1.

### 3.2. Clinical Features by Histological Categories

Profound heterogeneities in clinical variables were associated with histological categories of sarcoma (Figure 1B). The median age at diagnosis varied among different groups of patients, with those aged 18–34 years associated with Ewing sarcoma (819/1316, 62.23%), sarcoma with SBRC features (309/596, 51.85%), and osteosarcoma (1223/3144, 38.90%). By contrast, patients aged over 85 years were more likely associated with giant cell sarcoma/UPS (648/4953, 13.08%), angiosarcoma (9/72, 12.50%), and sarcoma NOS (2773/30,980, 8.95%).

De novo metastatic disease was relatively frequent in sarcoma with SBRC features (150/596, 25.17%), Ewing sarcoma (260/1316, 19.80%), and rhabdomyosarcoma (376/2439, 15.42%). The organ-specific preference at the diagnosis of de novo metastatic disease was also assessed among different histological categories. A relatively higher incidence of liver metastasis at diagnosis was observed in sarcoma with SBRC (53/596, 8.89%), stromal sarcoma (820/13,505, 6.10%), and leiomyosarcoma (623/16,574, 3.80%), while de novo lung metastasis was more frequently associated with sarcoma with SBRC (77/596, 12.92%), Ewing sarcoma (132/1316, 10.00%), and rhabdomyosarcoma (236/2439, 9.70%). Patients with brain metastasis at diagnosis of sarcoma mostly displayed the subtypes of sarcoma with SBRC (7/596, 1.17%), rhabdomyosarcoma (22/2439, 0.9%), and Ewing sarcoma (10/1316, 0.8%) compared to the others with the proportion of brain metastasis below 0.5%.

### 3.3. Survival Outcomes Across Histological Categories

**Figure 2:**
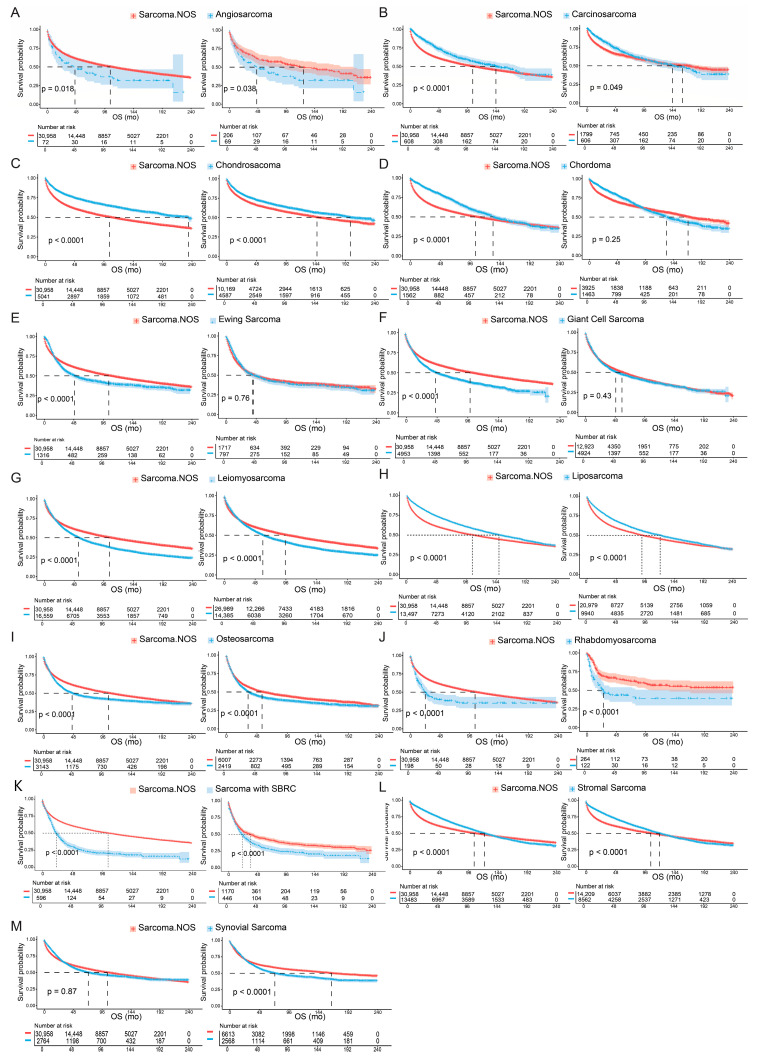
Comparative analysis of survival outcomes of overall population or patients upon propensity score matching. Pairwise comparisons of sarcoma NOS and the other histological subtypes were performed among overall population and the patients after 1:3 propensity score matching (PSM). For each group of comparisons (**A**–**H**), the result of overall patients is presented on the left and the result of PSM analysis is shown on the right. (**A**) Angiosarcoma; (**B**) Carcinosarcoma; (**C**) Chondrosarcoma; (**D**) Chordoma; (**E**) Ewing sarcoma; (**F**) Giant cell sarcoma; (**G**) Leiomyosarcoma; (**H**) Liposarcoma; (**I**) Osteosarcoma; (**J**) Rhabdomyosarcoma; (**K**) Sarcoma with small blue round cell (SBRC) features; (**L**) Stromal sarcoma; (**M**) Synovial sarcoma.

Regarding the survival outcomes, sarcoma with different histological subtypes presented distinctive prognostic profiles (Figure 1C). Patients with chondrosarcoma presented the longest median overall survival (mOS) of 234 mo (95% CI 200-NA mo), followed by liposarcoma (148 mo, 95% CI 142–156 mo), and carcinosarcoma (144 mo, 95% CI 114–180 mo). By contrast, the shortest mOS was demonstrated in rhabdomyosarcoma (16 mo, 95% CI 16–18 mo), sarcoma with SBRC features (20 mo, 95% CI 20–26 mo), osteosarcoma (46 mo, 95% CI 41–53 mo), and angiosarcoma (46 mo, 95% CI 23–109 mo). Comparative analyses of OS are listed at Table 1. Among primary sarcomas, rhabdomyosarcoma demonstrated the shortest median overall survival (mOS: 16 months, 95% CI 16–18 months), confirming its status as the deadliest histological subtype in this large population-based cohort. Notably, rhabdomyosarcoma consistently showed the worst prognosis regardless of whether it was classified as a primary or secondary malignancy, underscoring its aggressive clinical behavior and urgent need for improved therapeutic strategies. To account for potential confounding factors, we conducted a PSM analysis comparing each histological subtype to sarcoma NOS (Figure 2A–M). Post-PSM, most histologies remained significantly distinct in terms of OS. Notably, synovial sarcoma showed comparable OS to sarcoma NOS before matching but exhibited significantly worse OS after matching (*p* < 0.0001). In contrast, chordoma, Ewing sarcoma, and giant cell sarcoma/UPS maintained comparable OS relative to sarcoma NOS after adjustment.

Median OS (months) with 95% confidence intervals (CIs) is listed for each histological subtype. *p*-values indicate statistical significance from pairwise comparisons using the log-rank test. Comparisons are shown below the diagonal; values along the diagonal are shaded. Histological categories with significant differences in survival outcomes are highlighted, reflecting substantial heterogeneity in prognosis across histologies. We next explored prognostic factors across different subtypes (Tables S2–S15). Advanced age was consistently associated with poorer OS in nearly all sarcomas, with the exception of angiosarcoma and carcinosarcoma. Female sex was independently associated with improved survival in chondrosarcoma (HR 0.81, 95% CI 0.73–0.89; *p* < 0.001), liposarcoma (HR 0.85, 95% CI 0.80–0.90; *p* < 0.001), osteosarcoma (HR 0.83, 95% CI 0.75–0.92; *p* < 0.001), stromal sarcoma (HR 0.79, 95% CI 0.75–0.83; *p* < 0.001), and synovial sarcoma (HR 0.77, 95% CI 0.69–0.86; *p* < 0.001) but was associated with worse OS in leiomyosarcoma (HR 1.32, 95% CI 1.26–1.38; *p* < 0.001). While liver metastasis was more common in sarcoma with SBRC features and leiomyosarcoma, it did not independently affect OS. Conversely, lung metastases were adverse prognostic indicators in Ewing sarcoma (HR 1.51, 95% CI 1.13–2.02; *p* = 0.005), sarcoma with SBRC features (HR 1.72, 95% CI 1.18–2.48; *p* = 0.004), and rhabdomyosarcoma (HR 1.33, 95% CI 1.09–1.62; *p* = 0.004). Brain metastasis, although rare, independently predicted worse OS in multiple subtypes, including carcinosarcoma (HR 32.05, 95% CI 5.75–137.32; *p* = 0.001), chondrosarcoma (HR 4.09, 95% CI 1.77–9.43; *p* = 0.001), giant cell sarcoma/UPS (HR 1.82, 95% CI 1.13–2.94; *p* = 0.013), leiomyosarcoma (HR 1.50, 95% CI 1.08–2.07; *p* = 0.014), liposarcoma (HR 2.48, 95% CI 1.34–4.06; *p* = 0.004), rhabdomyosarcoma (HR 1.70, 95% CI 1.06–2.72; *p* = 0.027), sarcoma NOS (HR 2.30, 95% CI 1.86–2.84; *p* < 0.001), and synovial sarcoma (HR 3.42, 95% CI 1.64–7.15; *p* = 0.001).

### 3.4. Comparisons of Primary and Secondary Sarcomas

**Figure 3:**
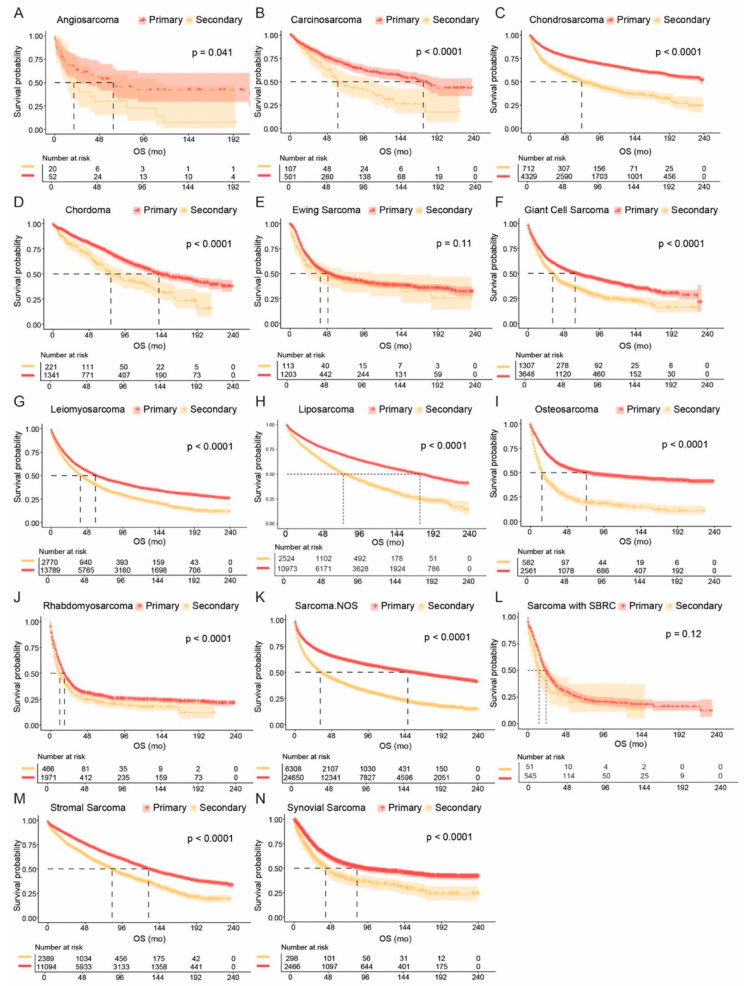
Comparative analysis of prognosis of primary sarcoma and secondary sarcoma with different histological subtypes. Kaplan–Meier survival curves depicting overall survival (OS) in patients with primary sarcoma (red) and secondary sarcoma (orange) stratified by histological subtype. Statistical significance was assessed using the log-rank test, and *p*-values are displayed on each plot. (**A**) Angiosarcoma; (**B**) Carcinosarcoma; (**C**) Chondrosarcoma; (**D**) Chordoma; (**E**) Ewing sarcoma; (**F**) Giant cell sarcoma; (**G**) Leiomyosarcoma; (**H**) Liposarcoma; (**I**) Osteosarcoma; (**J**) Rhabdomyosarcoma; (**K**) Sarcoma not otherwise specified (NOS); (**L**) Sarcoma with small blue round cell (SBRC) features; (**M**) Stromal sarcoma; (**N**) Synovial sarcoma.

We further compared outcomes between primary and secondary sarcomas (Figure 3 and Figure S2, Tables S16 and S17). Across nearly all histological subtypes, primary sarcomas were associated with significantly better OS than secondary tumors. The longest survival in primary disease was observed in chondrosarcoma (mOS not reached, 95% CI 234-NA mo), liposarcoma (175 mo, 95% CI 167–185 mo), and carcinosarcoma (mOS 174 mo, 95% CI 144-NA mo). Among secondary sarcomas, stromal sarcoma (mOS 83 mo, 95% CI 76–89 mo), chordoma (mOS 77 mo, 95% CI 68–113 mo), and liposarcoma (mOS 74 mo, 95% CI 69–83 mo) demonstrated the most favorable prognosis, while rhabdomyosarcoma (mOS 12 mo, 95% CI 10–15 mo), sarcoma with SBRC features (mOS 14 mo, 95% CI 10–26 mo), and osteosarcoma (mOS 15 mo, 95% CI 13–19 mo) showed the worst outcomes. Exceptions to this pattern included Ewing sarcoma (*p* = 0.11) and sarcoma with SBRC features (*p* = 0.12), where OS did not significantly differ between primary and secondary cases.

### 3.5. Treatment Patterns and Survival Outcomes

**Figure 4:**
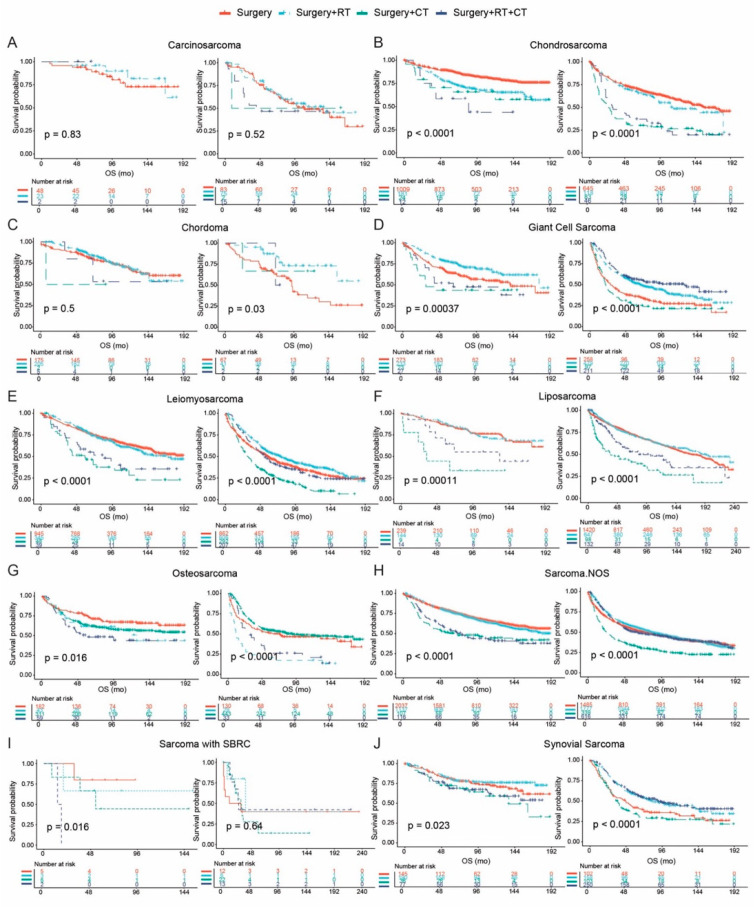
Survival outcomes associated with therapeutic options for operable sarcoma with different histological subtypes. Survival outcomes associated with treatment strategies for operable sarcomas by histological subtype and tumor size. Kaplan–Meier curves compare overall survival (OS) among patients receiving surgery alone, surgery + radiotherapy (RT), surgery + chemotherapy (CT), or surgery + RT + CT. For each subtype, patients were stratified by tumor size (≤5 cm on the left; >5 cm on the right). Subfigures present comparisons for the following sarcoma subtypes: (**A**) carcinosarcoma, (**B**) chondrosarcoma, (**C**) chordoma, (**D**) giant cell sarcoma, (**E**) leiomyosarcoma, (**F**) liposarcoma, (**G**) osteosarcoma, (**H**) sarcoma not otherwise specified (NOS), (**I**) sarcoma with small blue round cell (SBRC) features, and (**J**) synovial sarcoma. *p*-values are based on log-rank tests.

**Figure 5:**
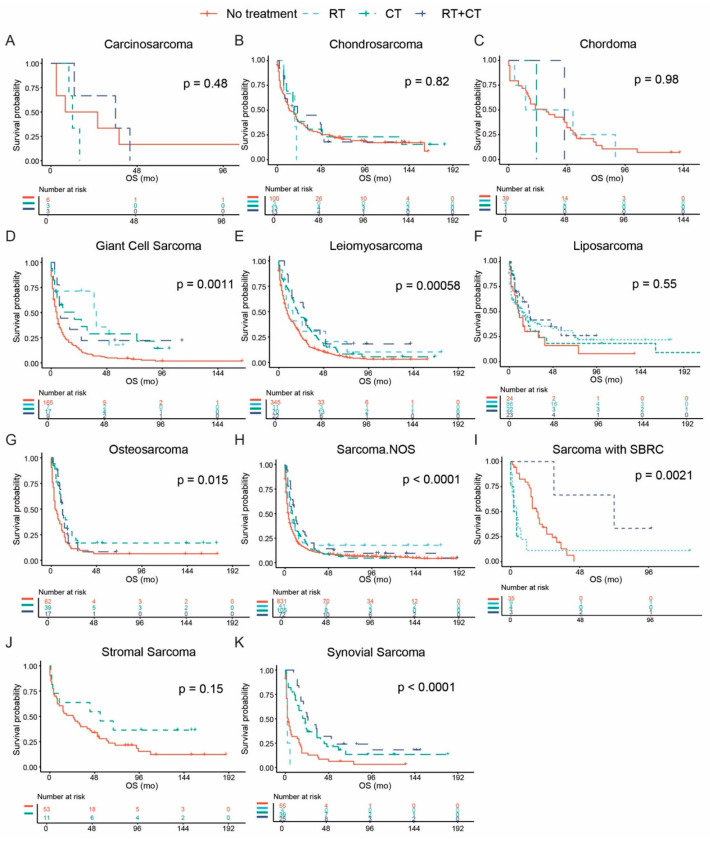
Survival outcomes associated with therapeutic options for inoperable sarcoma with tumor stage ≥ T2. Kaplan–Meier curves compare overall survival (OS) among patients who received no treatment, radiotherapy (RT) alone, chemotherapy (CT) alone, or a combination of RT + CT. Treatment effects were evaluated across histological subtypes, showing varied survival benefits depending on sarcoma type. Subfigures represent the following sarcoma subtypes: (**A**) carcinosarcoma, (**B**) chondrosarcoma, (**C**) chordoma, (**D**) giant cell sarcoma, (**E**) leiomyosarcoma, (**F**) liposarcoma, (**G**) osteosarcoma, (**H**) sarcoma not otherwise specified (NOS), (**I**) sarcoma with small blue round cell (SBRC) features, (**J**) stromal sarcoma, and (**K**) synovial sarcoma. *p*-values are based on log-rank tests.

Survival outcomes associated with different therapies were assessed among histological subtypes of sarcoma. We divided the tumors for the comparative analysis according to AJCC T stage, comprising T0/T1 and ≥ T2 subgroups (Figure 4 and Figure 5, Tables S18–S20).

Regarding operable disease, patients with giant cell sarcoma can obtain more OS benefits (*p* < 0.0001) with the receipt of surgery plus radiation therapy; the addition of chemotherapy only benefited those with tumor size > 5 cm (*p* < 0.0001) (Figure 4D). The combination of radiation therapy and surgery versus surgery alone demonstrated comparable mOS for patients with liposarcoma (Figure 4F). However, this combination treatment could significantly prolong the OS for patients with chordoma (*p* = 0.03) and leiomyosarcoma (*p* < 0.0001) with tumor size > 5 cm, in addition to synovial sarcoma with tumor size ≤ 5 cm (*p* = 0.023) (Figure 4C,E,J).

For inoperable disease with tumor size staged ≥ T2, chemotherapy only could be the better option for patients with sarcoma with SBRC (*p* = 0.0021). Similar results were observed among patients with osteosarcoma, for whom chemotherapy could significantly prolong the OS (*p* = 0.015); the divergence was caused by the fact that the addition of radiation therapy cannot further enhance this kind of survival benefit. Monotherapy could be a better choice than the combination treatment for giant cell sarcoma/UPS (*p* = 0.0011); however, no significant difference was detected between radiation therapy and chemotherapy.

## 4. Discussion

This study presents the most comprehensive population-level analysis to date that examines the clinicopathological characteristics and survival outcomes across multiple histological subtypes of sarcoma. Rather than focusing on a single entity or site, we provide a panoramic view of sarcoma heterogeneity using a large, real-world cohort. These findings underscore the complex biological and clinical diversity within sarcoma, which has long challenged oncologists and pathologists in both diagnosis and treatment [9,10].

A key strength of this study is its scale and inclusivity. By classifying sarcomas into 14 distinct histological categories, we attempted to bridge the gap between rare disease biology and actionable clinical insights. Importantly, this approach highlights how distinct histologies not only differ in metastatic behavior and demographic patterns but also in their response to standard treatments [11,12]. This heterogeneity, often underappreciated in clinical practice, should prompt a shift away from “one-size-fits-all” management toward a more subtype-specific framework. Historically, sarcomas have been managed as a unified entity in many clinical trials, potentially obscuring meaningful differences in response and outcome [13,14]. Our findings suggest that future trial designs and therapeutic algorithms should stratify or even exclusively target specific histologies, particularly those with aggressive metastatic profiles or unique treatment sensitivities [15,16].

Similarly, the organotropism of metastases observed across histologies hints at intrinsic molecular cues guiding tumor dissemination. The tendency for some sarcomas to preferentially metastasize to lung or liver might reflect differential expression of adhesion molecules, matrix-remodeling enzymes, or chemokine receptors [14]. While this study is unable to directly probe these molecular mechanisms, it lays the groundwork for future work integrating a clinical phenotype with genomic and transcriptomic data to decipher the metastatic logic of sarcoma [17].

One of the most clinically relevant takeaways is the need to revisit treatment paradigms across subtypes. While surgery remains the mainstay, the role of chemotherapy and radiotherapy is far more nuanced than current guidelines often acknowledge [18,19,20]. Our analysis suggests that treatment decisions should be more closely guided by tumor histology and size, rather than generalized staging criteria. The fact that combination regimens were beneficial in some inoperable subtypes but not others supports the idea that histology-informed regimens may yield a better therapeutic index [21,22]. Equally important is the finding that certain “chemosensitive” sarcomas do not always benefit from chemotherapy depending on stage or resectability, challenging conventional assumptions [23,24]. Moving forward, clinical guidelines would benefit from incorporating histology-specific evidence, potentially supported by dedicated prospective registries or adaptive trials focusing on rare cancers [25]. Moreover, the observed disparities in treatment patterns between subtypes—especially those impacting survival—highlight concerns regarding therapeutic access and potential physician bias. It is possible that some sarcoma subtypes are more likely to trigger aggressive or guideline-concordant care due to familiarity, whereas others may be undertreated due to rarity or diagnostic uncertainty [26,27]. This reflects a need for broader education and decision-support tools for oncologists managing rare sarcoma subtypes.

From a methodological perspective, the use of propensity score matching allowed us to better isolate histology-related prognostic effects by minimizing the influence of confounding variables such as age, sex, and treatment exposure. However, one should still interpret these comparisons with caution. Propensity matching improves comparability but cannot fully account for unmeasured variables—especially those not captured in SEER, such as performance status, comorbidities, or molecular features [28,29,30]. This highlights a broader limitation of population-based datasets, which, despite their size and generalizability, lack granularity in therapeutic intent, disease recurrence, and genomic profiling.

Importantly, this work also brings to light the challenge of classifying sarcomas in large databases. The histological groupings we adopted reflect a balance between clinical relevance and data constraints but inevitably simplify a biologically diverse landscape [31]. The SEER database relies on ICD-O histology codes, which may not fully align with current WHO classifications or reflect evolving molecular taxonomies [32]. For example, the category “giant cell sarcoma” is an outdated term, now classified under ICD-11 (code XH73J4) as synonymous with undifferentiated pleomorphic sarcoma or pleomorphic sarcoma, NOS. It is important to acknowledge that “giant cell sarcoma” is not recognized as a distinct histological entity in the current WHO classification. Additionally, SEER captures the dominant histological pattern, often overlooking biphasic or composite tumors, which could introduce misclassification bias—particularly in tumors with high heterogeneity, such as dedifferentiated liposarcoma or malignant peripheral nerve sheath tumors [33]. SEER also lacks data on fusion status and molecular profiles, which are now integral to the diagnosis and classification of many sarcoma subtypes. As a result, tumors with distinct molecular drivers—such as CIC-rearranged sarcoma or BCOR-rearranged sarcomas—may be clumped under broader morphological categories such as sarcoma with small round cell features. Given the evolving molecular classification of liposarcoma, we grouped round cell liposarcoma with other liposarcoma subtypes, recognizing it as a high-grade variant of myxoid liposarcoma. Although not without limitations—since myxoid liposarcomas are molecularly distinct entities from well-differentiated and dedifferentiated liposarcomas—this approach preserves biologically coherent groupings within the constraints of SEER, which lacks detailed molecular annotation. Sarcoma, NOS, which accounted for a large portion of the cohort, is likely an umbrella term encompassing a broad differential. These may include undifferentiated spindle cell sarcomas, sarcomatoid carcinomas or undifferentiated melanomas misclassified as sarcomas, and a spectrum of fusion-driven spindle cell sarcomas that have only recently been recognized. As such, interpretations involving this group should be approached with caution, given its diagnostic, biological, and molecular heterogeneity.

Beyond these structural limitations, SEER does not include information on novel therapies such as targeted agents or immunotherapies, which have become increasingly relevant for select sarcoma subtypes [9,34]. The omission of these treatments means our survival analyses primarily reflect outcomes associated with conventional therapy and may not capture recent progress in personalized treatment. For instance, tumors harboring NTRK fusions, ALK rearrangements, or TSC mutations—each now targetable—may behave differently than suggested by historical data [35,36,37].

While histology provides a valuable framework for prognostic stratification, the integration of molecular and genetic data is essential for fully realizing individualized therapy in sarcoma. For example, the identification of fusion genes such as EWSR1-FLI1 in Ewing sarcoma, SYT-SSX in synovial sarcoma, and amplification of MDM2 in liposarcoma have already reshaped classification and guided targeted therapy approaches [34,38,39]. Recent evidence suggests that, even within a single histological category, distinct molecular alterations can define biologically and clinically relevant subgroups [40]. In osteosarcoma, recent studies have revealed marked intratumoral heterogeneity, transcriptional subtypes, and chromosomal instability that correlate with clinical behavior and treatment response [41,42,43]. Most notably, a recent transcriptomic analysis by the Cancer Grand Challenges program revealed the transcriptional footprint of osteosarcoma using one of the largest global datasets, identifying new avenues for molecularly guided therapy [44]. Thus, future directions in sarcoma research and clinical care should aim for a harmonized approach that integrates histological patterns with molecular signatures to improve diagnostic precision and therapeutic outcomes [45].

## 5. Conclusions

This large-scale, population-based study provides a comprehensive overview of the clinicopathological heterogeneity and prognostic implications of distinct sarcoma histological subtypes. Our findings highlight substantial differences in age distribution, metastatic patterns, and survival outcomes across subtypes, underscoring the critical need for histology-specific risk stratification and treatment planning. While surgery remains a cornerstone of sarcoma management, the role of chemotherapy and radiotherapy varies significantly by histology and tumor stage, emphasizing the importance of individualized therapeutic strategies. Future efforts should integrate molecular profiling and prospective data collection to refine classification systems, guide treatment decisions, and improve outcomes for patients with this diverse and challenging group of malignancies.

## Figures and Tables

**Table 1:** Comparison of median overall survival (mOS) across histological subtypes of sarcoma.

Histology OS (95% CI), Months	Angiosarcoma 46 (23–109)	Carcinosarcoma 144 (114–180)	Chondrosarcoma 234 (200–NA)	Chordoma 133 (122–147)	Ewing Sarcoma 49 (41–58)	Giant Cell Sarcoma 49 (45–55)	Leiomyosarcoma 55 (53–57)	Liposarcoma 148 (142–156)	Osteosarcoma 46 (41–53)	Rhabdomyosarcoma 16 (16–18)	Sarcoma.NOS 105 (100–110)	Sarcoma with SBRC 22 (20–26)	Stromal Sarcoma 122 (117–125)	Synovial Sarcoma 73 (64–86)
Angiosarcoma 46 (23–109)														
Carcinosarcoma 144 (114–180)	<0.0001													
Chondrosarcoma 234 (200–NA)	<0.0001	0.039												
Chordoma 133 (122–147)	<0.0001	0.520	0.008											
Ewing sarcoma 49 (41–58)	0.249	<0.0001	<0.0001	<0.0001										
Giant cell sarcoma 49 (45–55)	0.668	<0.0001	<0.0001	<0.0001	0.029									_
Leiomyosarcoma 55 (53–57)	0.515	<0.0001	<0.0001	<0.0001	0.117	0.042								
Liposarcoma 148 (142–156)	<0.0001	0.890	<0.0001	0.484	<0.0001	<0.0001	<0.0001							
Osteosarcoma 46 (41–53)	0.323	<0.0001	<0.0001	<0.0001	0.449	0.118	0.053	<0.0001						
Rhabdomyosarcoma 16 (16–18)	0.015	<0.0001	<0.0001	<0.0001	<0.0001	<0.0001	<0.0001	<0.0001	<0.0001					
Sarcoma.NOS 105 (100–110)	0.022	<0.0001	<0.0001	<0.0001	<0.0001	<0.0001	<0.0001	<0.0001	<0.0001	<0.0001				
Sarcoma with SBRC 22 (20–26)	0.019	<0.0001	<0.0001	<0.0001	<0.0001	<0.0001	<0.0001	<0.0001	<0.0001	0.129	<0.0001			
Stromal sarcoma 122 (117–125)	<0.0001	0.199	<0.0001	0.002	<0.0001	<0.0001	<0.0001	<0.0001	<0.0001	<0.0001	<0.0001	<0.0001		
Synovial sarcoma 73 (64–86)	0.023	<0.0001	<0.0001	<0.0001	<0.0001	<0.0001	<0.0001	<0.0001	<0.0001	<0.0001	0.883	<0.0001	<0.0001	

NA, Not available.

## Data Availability

The original data presented in this study are openly available in the Surveillance, Epidemiology, and End Results (SEER) database at https://seer.cancer.gov (accessed on 16 May 2025). Access to the SEER database requires registration and compliance with the SEER data use agreement.

## Supplementary Materials

The following supporting information can be downloaded at: https://www.mdpi.com/article/10.3390/cancers17101706/s1, Figure S1. Patient selection and eligibility. Figure S2. Survival outcomes of primary (A) and secondary (B) sarcoma. Table S1. Baseline Demographic and Clinical Characteristics of All Sarcoma Patients. Table S2. Clinicopathological Features of Sarcoma Not Otherwise Specified. Table S3. Clinicopathological Features of Angiosarcoma. Table S4. Clinicopathological Features of Carcinosarcoma. Table S5. Clinicopathological Features of Chondrosarcoma. Table S6. Clinicopathological Features of Chordoma. Table S7. Clinicopathological Features of Ewing Sarcoma. Table S8. Clinicopathological Features of Giant Cell Sarcoma (Undifferentiated Pleomorphic Sarcoma). Table S9. Clinicopathological Features of Leiomyosarcoma. Table S10. Clinicopathological Features of Liposarcoma. Table S11. Clinicopathological Features of Osteosarcoma. Table S12. Clinicopathological Features of Rhabdomyosarcoma. Table S13. Clinicopathological Features of Sarcoma with Small Blue Round Cell Features. Table S14. Clinicopathological Features of Stromal Sarcoma. Table S15. Clinicopathological Features of Synovial Sarcoma. Table S16. Overall Survival Outcomes in Primary Sarcoma. Table S17. Overall Survival Outcomes in Secondary Sarcoma. Table S18. Treatment Outcomes in Operable Tumors <5 cm. Table S19. Treatment Outcomes in Operable Tumors ≥5 cm. Table S20. Treatment Outcomes in Inoperable Tumors ≥5 cm.
