# Supplementary material for: Clinical Differences Among Histological Categories of Sarcoma: Insights from 97,062 Patients
Source: Cancers (Basel). 2025 May 20;17(10):1706. doi: 10.3390/cancers17101706 (PMC12109841; doi:10.3390/cancers17101706)

Supplementary Figures

FigureS1. Patient selection and eligibility

NOS, Not otherwise specified.

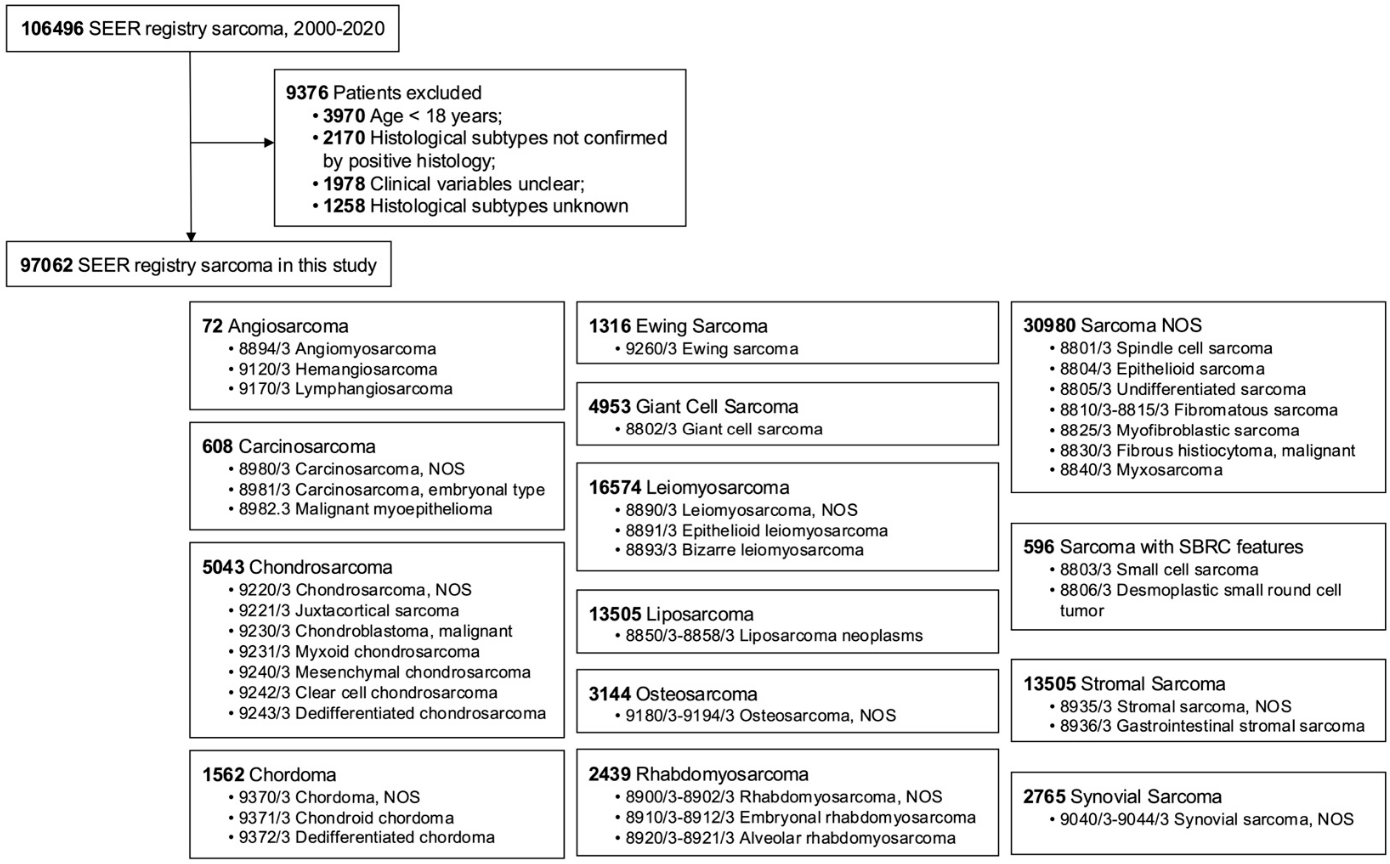

FigureS2. Survival Outcomes of Primary (A) and Secondary (B) Sarcoma

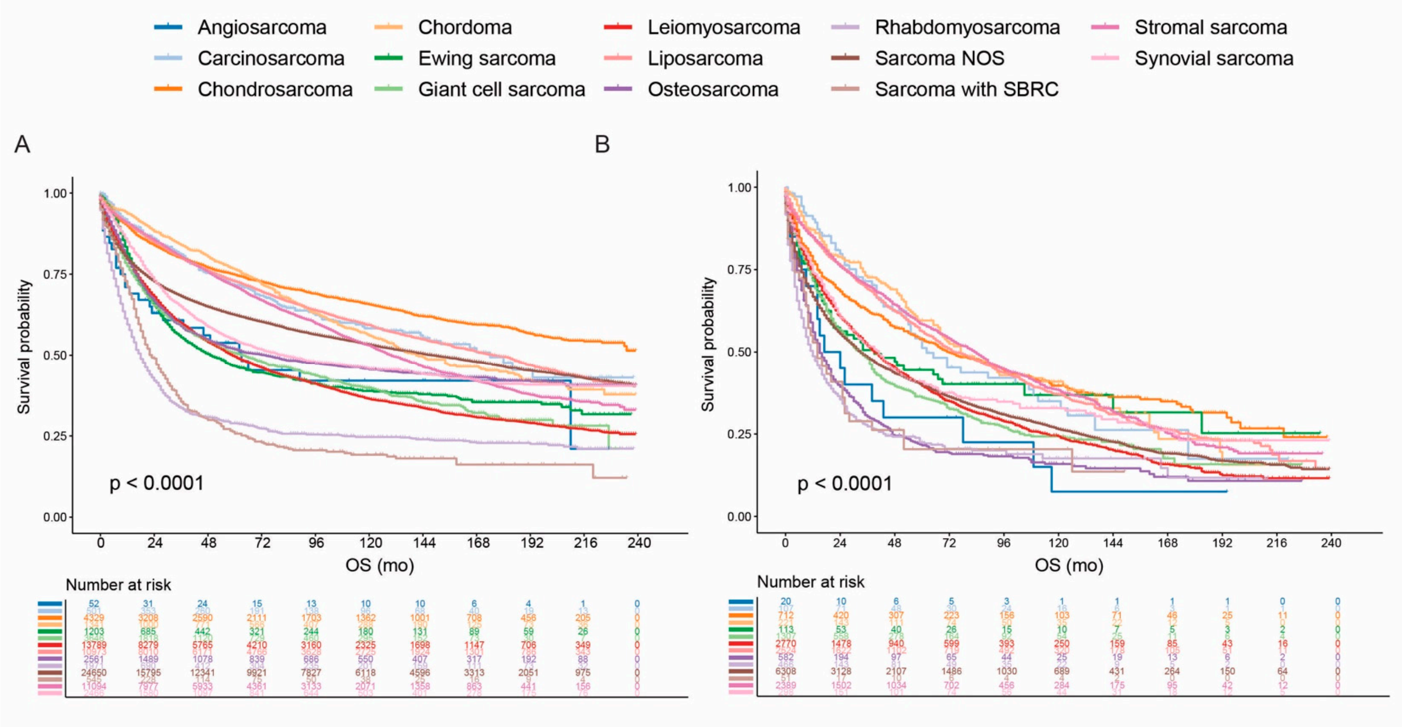

Supplement: Supplementary file 1 [file cancers-17-01706-s001.zip › Suppl Figures.pdf]
